# Farming system effects on root rot pathogen complex and yield of faba bean (*vicia faba*) in Germany

**DOI:** 10.3389/fpls.2022.1009906

**Published:** 2022-12-21

**Authors:** Adnan Šišić, Jelena Baćanović-Šišić, Harald Schmidt, Maria R. Finckh

**Affiliations:** ^1^ Department of Ecological Plant Protection, University of Kassel, Witzenhausen, Germany; ^2^ Section of Organic Plant Breeding and Agrobiodiversity, University of Kassel, Witzenhausen, Germany; ^3^ Foundation Ecology & Agriculture (SOEL), Ahrweiler, Germany

**Keywords:** fusarium, didymella, root rot, faba bean, grain legumes, organic agriculture, conventional agriculture

## Abstract

A survey across Germany was undertaken from 2016-2019 to evaluate effects of management system (organic vs conventional), pedo-climatic conditions and crop rotation history on faba bean root health status, diversity of major root rot pathogens and yield. Root rot incidence was generally low and there was no effect of the management system on the spectrum of pathogens isolated. Among the most common fungal species identified, frequencies of *Fusarium redolens* and *Didymella pinodella* were significantly higher in roots from organic fields compared with conventional and lower was observed for *F. avenaceum*, *F. tricinctum* and *F. culmorum*. Faba bean roots were colonized at similar rates by *F. equiseti* and the members of the *F. oxysporum* (FOSC) and *F. solani* (FSSC) species complexes in both management systems. Almost no legumes had been grown in the 5-11 years preceding the conventional faba beans surveyed while legumes had almost always been present during this period in the organic fields. This difference in rotational histories between the farming systems led to apparent cropping systems effects on the isolation frequencies of several species. For example, *D. pinodella* was ubiquitous in organic fields with a high frequency of legumes in the rotations but much rarer and often absent in conventional fields. Pedo-climatic conditions, particularly cool conditions at sowing and plant emergence and/or during the vegetative season favored most of the most prevalent *Fusarium* species identified in this study. In organic systems, yields correlated negatively with *D. pinodella* and *F. redolens* frequencies whereas higher levels of *F. tricintum* in faba bean roots had a positive correlation with yield. In conventional systems, faba bean yields depended more on the total precipitation before sowing and during the main growing season but were also negatively correlated with the frequencies of FOSC and *F. culmorum*. Phylogenetic analysis based on the *TEF1 alpha* locus indicated that the FSSC isolates mainly belonged to the *F. pisi* lineage. In contrast, the FOSC isolates were placed in 9 different lineages, with a conspicuous dominance of *F. libertatis* that has until now not been associated with any leguminous host.

## Introduction

Foot and root rots, caused by a complex of soil-borne pathogens are among the most widespread and important grain legume diseases and one of the major constrains in grain legume production worldwide (Wille et al., 2019). Several *Fusarium* species, *Aphanomyces euteiches* and the *Ascochyta* complex pathogens, *Didymella pinodella* and *D. pinodes*, are the most commonly associated pathogens with the disease complex (Baćanović-Šišić et al., 2018; Wille et al., 2019). Plants under field conditions are usually colonized by multiple pathogens simultaneously, and the importance of each species varies depending on the geographical region, pedo-climatic conditions and the crop management strategy (Esmaeili Taheri et al., 2016; Naseri and Ansari Hamadani, 2017; Baćanović-Šišić et al., 2018; Chatterton et al., 2019; Williamson-Benavides and Dhingra, 2021). In northern USA and Canada for example, *F. avenaceum* together with *Aphanomyces eutheiches* is a major threat to pea and lentil production (Chittem et al., 2015; Chatterton et al., 2019), whereas in France and northern European countries including Denmark and Sweden, in addition to *A. euteiches* and *F. avenaceum*, *F. solani*, *F. redolens* and *Didymella pinodella* (syn. *Phoma medicaginis* var. *pinodella*) play an important role in pea growing areas (Persson et al., 1997; Hossain et al., 2012; Gibert et al., 2022). Other pathogens such as, *Rhizoctonia solani*, *Pythium* spp., *Thielaviopsis basicola* and *Macrophomina phaseolina* have also been implicated as important parts of the grain legume root rot complex (Naseri and Mousavi, 2015; Wille et al., 2019; Williamson-Benavides and Dhingra, 2021; Wohor et al., 2022).

In the past 15 years, two large root rot surveys have been conducted on grain legumes in Germany. The first (2005-2007) focused on root health assessments of conventional peas, including detailed identification of the pathogens involved based on morphology (Pflughöft et al., 2012). This survey indicated declining importance of *F. solani* and *F. oxysporum* while *D. pinodella* together with *F. redolens* and *F. avenaceum* were identified as the primary pathogens in the pea root rot complex in Germany. The second survey (2008-2012) was conducted in four regions of Germany and, in addition to pea, also included faba beans but covered organic fields only (Wilbois et al., 2013). This survey focused solely on rating of the plants in the fields and, besides pointing to the importance of *Fusarium* spp. did not identify the major *Fusarium* species involved in the root rot complex of organic pea and faba bean. Although faba bean usually appeared healthier than pea they frequently harbored the same pathogens as peas (Pflughöft et al., 2012; Wilbois et al., 2013). No information is available on pathogens associated with conventional faba bean in Germany. Since the pathogen complex as a whole has a wide host range among legumes and, in addition, especially *Fusarium* spp. often affect cereals (Bainard et al., 2017; Walder et al., 2017), farmers are forced to grow pulses in wide rotations in order to avoid disease problems.

As a result of the new protein crop strategy adopted in 2012 by the German Federal Ministry of Food and Agriculture, and the EU Common Agricultural Policy (CAP) greening measures in 2015, faba bean production area in Germany has quadrupled since 2014 to about 60.000 ha in 2020. To avoid inoculum build-up and maintain or increase the area grown to faba bean and pulses in general, there is a need to evaluate current pathological risks in order to plan rotations. The overall objective of this study was to determine the root health status of organic and conventional faba beans in Germany and to characterize diversity and frequency of root associated pathogens as affected by farming system, pedo-climatic conditions and crop rotation management. We further report on the effects of root health status and root infections with major fungal species on faba bean yield and provide insights into the genetic variability of the *F. oxysporum* and *F*. *solani* isolates recovered.

## Material and methods

### Weather data, cropping history and site characteristics

**Figure 1:**
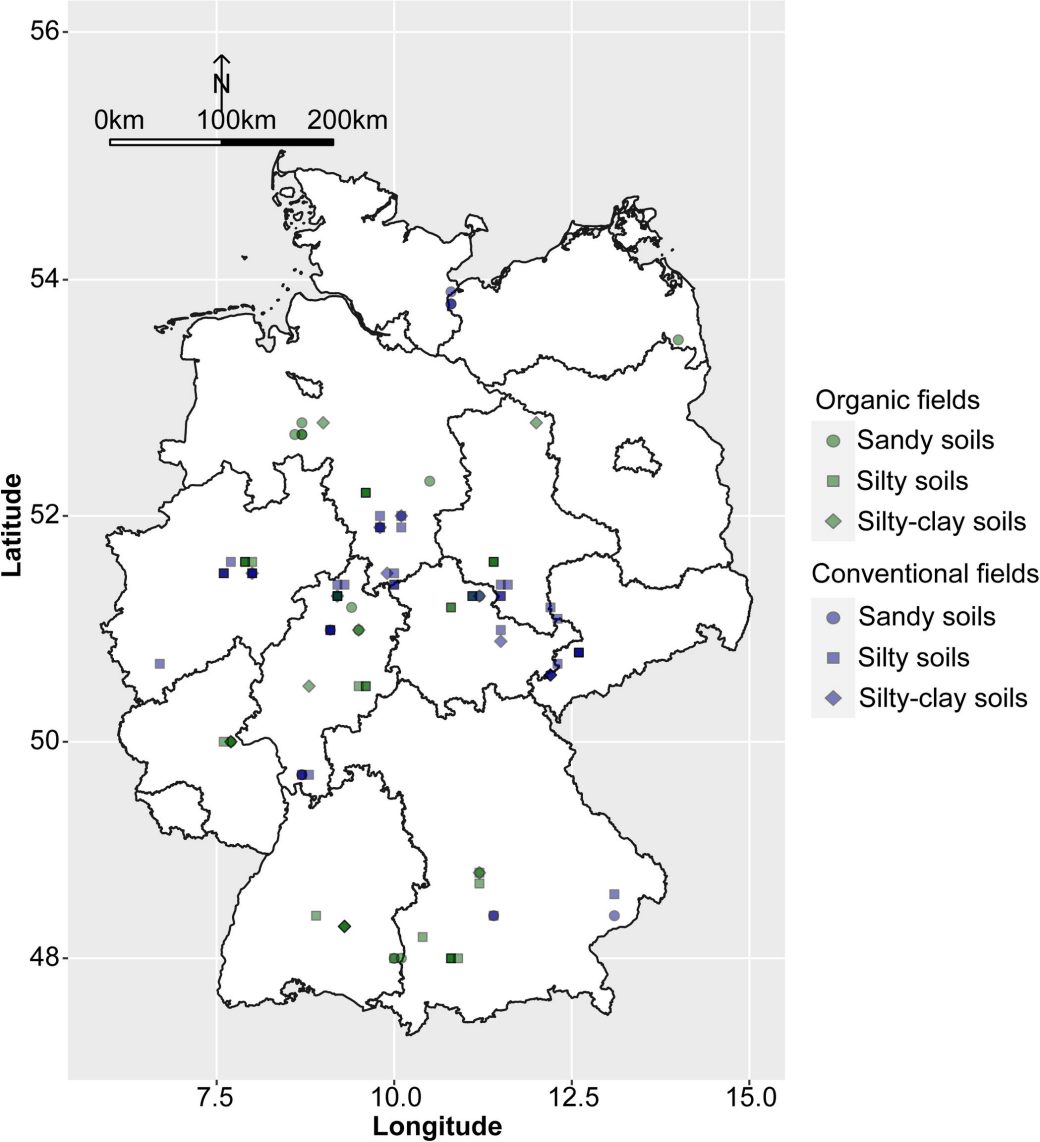
Map of Germany showing locations and the soil types of the surveyed organic and conventional faba bean fields.

Between 2016 and 2019, a total of 110 faba bean fields were sampled throughout Germany (**Figure 1**). Of these, 53 surveyed fields were managed organically according to the European Union and national standards and 57 fields conventionally. The meteorological data were obtained from the closest weather stations (<10 km) to the fields. Sand, silt, clay, and soil organic matter content as well as pH were determined in accordance with the standard DIN EN ISO/IEC 17025:2018-03. Data on cropping history were obtained from the farmers directly. These included the number of years fields were planted to different leguminous species (i.e. clover species and alfalfa, pea, faba bean, lentil, lupin, soybean, vetch and the unspecified group of ‘other grain’ or ‘small seeded legumes’) and the number of years of cereals (bulked data for all cereal crops) for a 5- and 11-year period prior to the sampling of faba bean. The complete data set is given in **Supplementary Table 1**.

### Sampling, disease assessments, morphological characterization of the isolates and yield estimation

**Figure 2:**
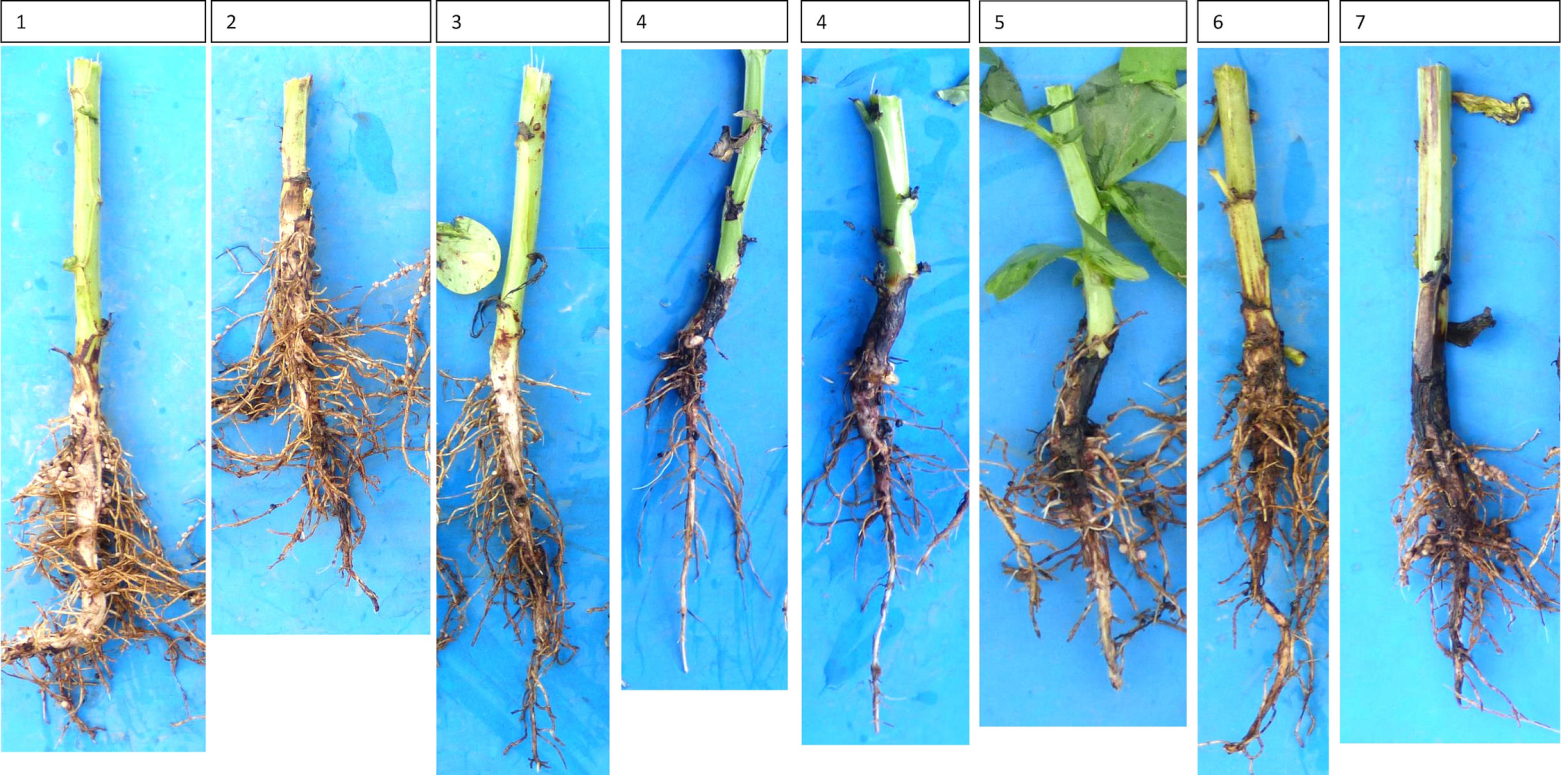
Varying root discoloration levels and the assigned root rot disease severity ratings (1=healthy plant, 9=dying plant). Plants with disease severity ratings of 8 and 9 (i.e. severe rots) were not observed during the survey.

Thirty-six to 40 faba bean plants were uprooted at full flowering from two areas per field, each 5 m^2^ in size with the distance between the two areas of 10 to 20 meters, depending on the field. Half of the roots were immediately washed to remove adhering soil and individual plants were evaluated for the severity of root rot symptoms using a visual 1-9 score (1=healthy, 9=dying plant) based on external root tissue discoloration levels (**Figure 2**) according to Pflughöft et al. (2012). The remaining half of the sampled roots were shipped to the University of Kassel and stored at -18°C until fungal isolations were performed as described previously (Šišić et al., 2018b). Isolations were targeted at the species belonging to the genus *Fusarium* and those sharing *Didymella* (*Phoma*) like morphology as these had been identified previously as the most common pathogens associated with field peas and faba beans in Germany (Pflughöft et al., 2012; Wilbois et al., 2013; Baćanović-Šišić et al., 2018). Briefly, roots were thoroughly washed under running tap water, surface sterilized with 3% sodium hypochlorite for 10 s, rinsed in distilled water and placed on filter paper under a laminar flow hood for ≥1 h. Three approximately 1-cm-long pieces per plant, representing root, crown, and the transition zone, were placed on COONs (Coons, 1916) media and incubated at 20˚C under 12 h cycles of UV light and dark. Roots included both lateral and tap roots up to the point of seed attachment, the crown was considered as the point of seed attachment up to ca. 0.5 cm below the soil surface and the transition zone covered about 1.5 cm between crown and stem.

After 1 to 2 weeks incubation, fungal colonies developing from the root pieces were sub-cultured separately in Petri dishes containing half-strength potato dextrose agar (19 g/l Difco PDA and 10 g/l agar). Pure cultures were generated either through hyphal tipping (*Fusarium* morphology) or transfer of single pycnida (*Didymella* morphology). Each isolate was examined microscopically and identified to the species level based on cultural appearance (colony color and pigmentation) and morphology of conidiogenous cells following the protocols of Leslie and Summerell (2006) for *Fusarium* spp. and Boerema et al. (2004) for *Didymella* spp.

Grain yield was estimated by hand harvesting the plants in five 0.5 m² plots within each of the 5 m^2^ area used for root collection. The yield was adjusted to 86% dry matter.

### Molecular confirmation of fungal species identity and phylogenetic analyses

The identity of 120 *Fusarium* and 8 *Didymella* randomly selected isolates obtained in this study representing 11 different fungal species (**Supplementary Table 2**) was confirmed by sequencing the portion of the translation elongation factor 1 (*TEF1*) alpha for *Fusarium* spp. (O’Donnell et al., 1998) and the beta tubulin gene region for *Didymella* spp. (Chen et al., 2015). DNA extraction, PCR amplification, sequencing and raw sequence data analysis were performed as described previously (Šišić et al., 2018a). Briefly, genomic DNA was extracted from pure cultures growing on half strength PDA agar plates (*Fusarium* spp; ½ strength PDA; 19 g/l Difco PDA and 10 g/l agar) or Coons (*Didymella* spp.; Coons, 1916) medium using the protocol described by Doyle and Doyle (1987). A portion of the translation-elongation factor 1 alpha (*tef1*) gene was amplified using primer pairs EF1 and EF2 (O’Donnell et al., 1998). The β tubulin (tub2) gene region was amplified with the primers Btub2Fd and Btub4Rd (Chen et al., 2015). Amplicons were visualized *via* electrophoresis on a 1% agarose gel and purified using the DNA Clean & Concentrator kit (Zymo Research, Freiburg, Germany) according to the manufacturer’s instructions. Sanger sequencing in both directions was performed by Macrogen Europe Laboratories (Amsterdam, Netherlands). Obtained row sequence data were assembled and errors identified and corrected manually in SeqMan Lasergene software (DNAStar, Madison, WI, U.S.A.). To confirm the taxonomic identity of the isolates, these sequences were used as queries for the *Fusarium*-ID v. 1.0 (Geiser et al., 2004) and NCBI (Madden, 2002) databases.

### Phylogenetic analyses

Single locus phylogenetic analyses based on the *TEF1 alpha* gene sequences were performed for the 35 *Fusarium oxysporum* species complex (FOSC) and 33 *Fusarium solani* species complex (FSSC) isolates. Reference sequences for the analysis were selected based on the previously published phylogenetic relationship within the FOSC (O’Donnell et al., 2009; Lombard et al., 2018) and the FSSC (O’Donnell et al., 2020; Geiser et al., 2021) (**Supplementary Tables 3** and **4**). The sequence alignments were generated using MAFFT v.7 (Katoh and Standley, 2013) and further adjusted manually with MEGA v6 (Tamura et al., 2013). A bootstrapped Maximum-Likelihood (ML) analysis was performed using the RAxML-VI-HPC v. 7.0.3 with non-parametric bootstrapping and 1000 replicates implemented on the Cipres portal (Stamatakis et al., 2008). For the FOSC we first performed the phylogenetic analysis on the data set which included 174 representative isolates from the study of O’Donnel et al. (2009) and 91 isolates from Lombard et al. (2018). The resulting *TEF1 alpha* tree topology revealed several isolates which belonged to the same forma specialis, shared identical or had similar *TEF1 alpha* gene sequences. These were excluded from subsequent analysis resulting in a data set which comprised 181 FOSC sequences. The FSSC data set consisted of 96 *TEF1* sequences (**Supplementary Tables 2**–**4**). For outgroup purposes, *F. udum* (CBS 177.31) and *F. thapsinum* (H05-557S-1 DCPA) were used to generate the phylogenetic trees.

### Statistical analyses

All statistical analyses were performed in R (R Core Team, 2013). Prior to the analysis, the abundance of individual fungal species was used to calculate isolation frequencies (i.e., percent colonized roots) by dividing the number of roots in which the species occurred by the total number of roots processed. The fields were further scored as positive or negative for presence of a particular fungal species and these data were used to calculate the prevalence for each pathogen in each management system (organic and conventional) by dividing the number of fields in which each species was present by the total number of fields sampled. In addition, the root rot incidence for each management system was calculated as the percentage of fields with mean disease severity score greater than 3 i.e., roots with clearly visible symptoms (**Figure 2**).

In the analysis of the isolation frequencies of individual fungal species associated with faba bean roots, rare species (i.e., <2% of total isolations) were not considered. To determine if the isolation frequencies were affected by the management system or sampling year, a generalized linear mixed model analysis was performed on proportional data with a binomial distribution and logit link function (Brooks et al., 2017). Fields were used as random effects, and to account for the two sampling areas within each field, sampling replicates were nested within. In data analyzed across sampling years, year was also used as random effect. Prevalence of fungal species and root rot incidence (the proportion of fields with mean disease severity ratings > 3; Chatterton et al., 2019) were treated as binary data (presence/absence) and were analyzed with Bayesian generalized linear model and logit link function (package ‘arm’, Su et al., 2018). The goodness of fit of the models was assessed using Pearson chi-square residual tests and further verified by the Kolmogorov–Smirnov test of normality and by checking if the data contain potentially significant outliers (package ‘DHARMa’, Hartig, 2021). Data were also visually inspected for normality by plotting the Pearson residuals against the expected values (package ‘ggplot2’, Wickham, 2016). The significance of the main effects in generalized linear models was assessed using an ANOVA function with the type III margin sum of squares (package ‘arm’). If significant treatment effects were observed, comparisons of least squares means with Tukey’s correction across the effect levels were performed (P < 0.05) (package ‘lsmeans’, Lenth, 2016).

Root rot severity data were analyzed with the non-parametric ranking procedure of the Kruskal-Wallis test (Conover, 1999) using the package ‘agricolae’ (Mendiburu, 2014). Management system and/or year were included as main explanatory variables. If significant treatment effects were observed (P<0.05), mean rank values were separated with the Kruskal multiple comparison test. For both, Kruskal-Wallis test and Kruskal multiple comparison test, the Benjamini and Hochberg (Benjamini and Hochberg, 1995) stepwise adjustment of P-values was used to control false discovery rate (FDR) and reduce type I errors.

The relationship between frequencies of individual fungal species and root rot incidence data including pedo-climatic, crop rotation and yield effects were examined using path analysis (package ‘lavaan’, Rosseel, 2012). Prior to the analysis, highly correlated environmental variables (Pearson r ≥ ± 0.7) were removed. Data were then subjected to the stepwise forward selection procedure (package ‘stats’, R Core Team, 2013) and only significant variables were retained in the path analysis. In addition, distinct soil clusters of the sampled fields were used as entries clustered based on their similarities in soil abiotic properties (sand, silt, clay, soil organic matter content and pH) employing the hierarchical clustering on principle components (HCPC) (package ‘FactoMineR’, Lê et al., 2008).

## Results

### Environmental conditions of the fields sampled

The 110 organic and conventional faba bean fields sampled represented a wide range of environments with respect to soil and climatic conditions (**Table 1**). Organic and conventional fields were placed in soil types ranging from sandy to loamy; large variation in soil organic matter (SOM) contents and moderate ranges in pH were present in organic and conventional fields.

**Table 1:** Range of pedo-climatic conditions for the faba bean fields sampled from 2016-2019.

	Organic (N=53)^a^			Conventional (N=57)		
Parameter	Minimum	Maximum	Median	Minimum	Maximum	Median
% sand	0.15	78.0	21.3	3.5	61.0	16.3
% silt	16.8	76.9	53.7	28.6	75.2	59.7
% clay	3.8	48.0	21.65	10.4	54.4	19
pH	5.1	7.3	6.55	5.9	7.3	6.7
Soil organic matter content (SOM)	1.5	4.9	2.6	1.8	4.8	2.6
Precipitation mm (14 days prior to sowing)	0	59	13.5	0	44	15
Precipitation mm (sowing-sampling)	21	517	167.6	48	327	174.6
Average temp. °C (01. Jan-sowing)	-0.4	5.5	2.3	0.0	4.8	2.3
Number of days <0° in March	0	10	0	0	11	0
Average temp. °C (14 days prior to sowing)	-3.8	9.7	6.4	-2.0	13.6	6.2
Average temp. °C (sowing-sowing + 14 days)	3.2	14.8	7.9	1.6	14.7	8.2
Average temp. °C (sowing-sampling)	7.1	16.8	13.3	10.1	17.3	12.8
Temp. sum °C (sowing-sampling)	902	1788	1316.2	959	1567	1309.7
Cereals (5 year history)^b^	1	5	3	0	5	3
Grain legumes (5 year history)^c^	0	2	0	0	1	0
Distance (grain legume crop)^d^	1	≥11	3	2	≥11	11

^a^ N=Number of fields.

^b^ Number of cereals grown during the 5 years preceding the sampling.

^c^ Number of times grain legumes were grown during the five years preceding the sampling.

^d^ Number of years since a grain legume crop was grown in the field prior to the sampling. No data beyond 11 years were available.

Sowing conditions ranged from very wet (up to 59 mm of rain in the 2 weeks before sowing) to no rain during the same period and, from very cold soils (minimal mean temperature two weeks before sowing -3.8°C) to very warm (maximal mean temperature before sowing 13.8°C). The driest conditions were observed in the year 2018 with a field receiving as little as 21 mm, the wettest in 2016 with a field that received 517 mm of rain between sowing and sampling (**Table 1** and **Supplementary Table 1**).

**Figure 3:**
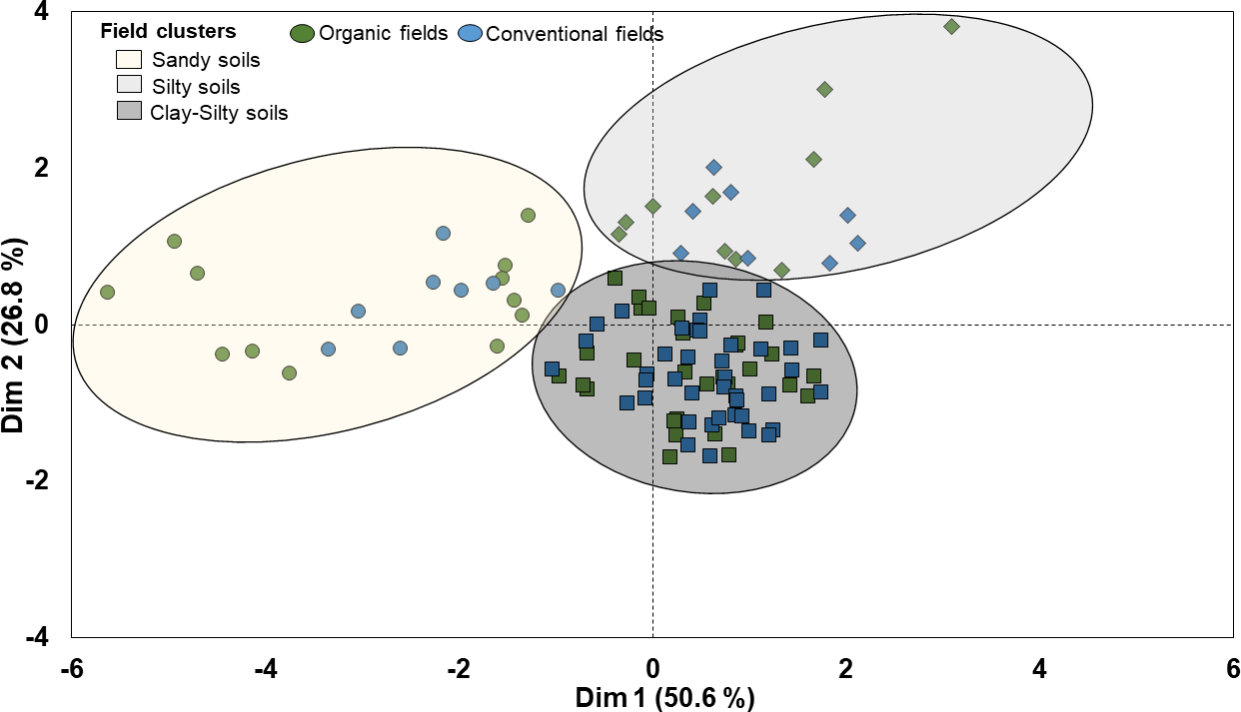
Cluster analysis for 110 organic and conventional faba bean fields based on their similarities in soil abiotic properties.

The hierarchical clustering of faba bean fields on principal components (HCPC) based on their similarities in soil parameters *i.e*. soil pH, sand, silt, clay and organic matter content (SOM) grouped the 110 faba bean fields into three clusters (cluster I, II and III) (**Figure 3**). The first two dimensions of the PCA summarized 77% of the variability in the data. Dimension 1 explained 50.6% of the variance and separated fields in cluster I from fields in clusters II and III based on their differences in soil pH values, sand and silt content. Dimension 2 explained 26.8% of the variance and separated the clusters mainly based on their differences in SOM content, which was also positively correlated with the soil clay content (e.g. cluster III) (**Figure 3** and **Table 2**). The PCA dimension 3 explained an additional 14.9% of the variability in the data and was most strongly related to the pH (not shown).

**Table 2:** The number of organic and conventional fields in each year, grouped by their similarities according to soil abiotic properties.

		Year^a^										
System	Cluster	2016	2017	2018	2019	N^b^	N^c^	SOM (%)^d^	pH	Clay (%)	Sand (%)	Silt (%)
Organic	I	4	4	2	2	12	218	2.3	6.0	12.1	55.6	32.3
	II	5	7	9	9	30	508	2.4	6.7	21.1	17.5	61.4
	III	3	3	5	0	11	220	3.7	6.4	31.4	18.2	50.4
Conventional	I	1	3	2	2	8	140	2.4	6.2	15.9	48.0	36.1
	II	10	10	8	12	40	675	2.6	6.8	18.8	16.2	65.0
	III	3	2	4	0	9	178	3.6	6.8	32.7	14.4	53.0

^a^ Number of fields sampled in 2016, 2017, 2018 and 2019.

^b^ N= Total number of fields.

^c^ n=Number of roots evaluated.

^d^ Mean value of soil organic matter content.

Cluster 1 included 12 organic and 8 conventional fields classified as sandy loam and characterized by lower pH (around 6.0) compared with the clusters II and III (around 6.7), and mean SOM content of 2.3% and 2.4% in organic and conventional fields, respectively (**Figure 3** and **Table 2**). Cluster II included 30 organic and 40 conventional fields associated with silty soils with higher pH (around 6.7) but similar SOM content as cluster I (**Figure 3** and **Table 2**). Cluster III included 11 organic and 9 conventional fields. It included silty clay soils but with higher clay and SOM (around 3.6%) contents than cluster II (SOM ca. 2.4%) (**Figure 3** and **Table 2**).

### Cropping histories

Across all fields for the 5-year period prior to the faba bean sampling, approximately 26% of the crops in rotations under organic management were legumes. Of these, approximately 70% were clover and alfalfa and about 30% grain legumes (mainly peas and faba beans - see below; hereafter grain legumes). In contrast, legumes in conventional fields constituted only about 5% of the crops in the 5-year rotation plan. The ratio of cereals in organic and conventional crops rotations was similar and accounted for 57% (organic) and 68% (conventional) of all crops. Overall frequencies of legumes and cereals for the 11-year period in both management systems was similar to the 5 year rotation plan (**Supplementary Table 5**).

**Figure 4:**
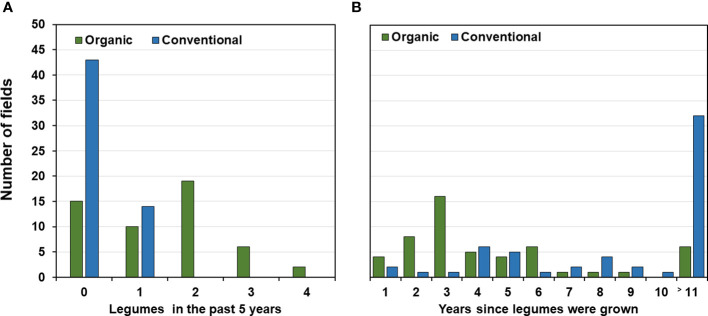
Ratio of legumes in organic and conventional crop rotations. **(A)** Number of fields and years since legumes were grown before the faba bean sampling. No information available for 11 or more years. **(B)** Number of fields depending on the frequency of legumes planted for the five-year period preceding the faba bean sampling.

In the organic fields, 37 out of the 52 (71%) fields for which data were available had been cropped with legumes either as main or cover crops during the past five years (**Figure 4A**). Clover and alfalfa had been grown in 32 (62%) fields usually for one to two years and grain legumes in 17 (32%) fields for one or two seasons. Also, cereals were part of the rotation during the preceding 5-years usually for two to three seasons (73% of the organic fields; **Supplementary Table 6**). In contrast, 43 (75%) of the conventional fields had not been planted to any legume in the preceding 5 years (**Figure 4A**). Twelve of the remaining 14 fields (25%) had been planted once with faba bean, one with pea during that period and one with small seeded legumes. Most rotations (48 out of 57 fields; 84%) included three or four years of cereals and five fields even 5 years while one conventional field had not been grown to cereals (**Figure 4B** and **Supplementary Table 6**). When considering the past 11 years prior to faba bean sampling, almost all organic farmers had grown legumes at least once while 32 of the 57 conventional farmers had not grown legumes during the least 11 years or longer (**Figure 4B**).

### Root rot incidence and root health status

Overall disease severity ratings (DSR) did not significantly differ between management systems (mean DSR of 2.6 and 2.0 for organic and conventional system, respectively) or among years (P≥0.28). However, there was a difference in the proportion of organic and conventional fields with clearly visible symptoms of root rot (i.e. root rot incidence, mean DSR>3). Faba bean plants from 18 out of 53 organic fields (34%) had mean DSR>3, significantly (P=0.03) more than in the conventional fields (9/57, 16%) with some variation among years (**Table 3**). Nevertheless, even in the organic fields, mean root rot severity was usually just above the threshold level of 3 with no significant difference in overall root rot symptom severity between the management systems (fields with mean DSR>3; **Table 3**).

**Table 3:** Mean root rot incidence (%) and root rot severity ratings (DSR) of faba bean fields in Germany, 2016-2019.

	Organic				Conventional				Overall	
Sampling year	2016	2017	2018	2019	2016	2017	2018	2019	Organic	Conventional
Root rot incidence (%)	58 a	21 bcd	50 ab	0 d	36 abc	0 d	29 abcd	0 d	34 a	16 b
Root rot severity^a^	3.9 ns	3.7	3.9	-^d^	3.6	–	3.6	–	3.9 ns	3.6
N^b^	12	14	16	11	14	15	14	14	53	57
n^c^	236	274	320	220	273	293	279	280	1050	1125

^a^Mean root rot severity for fields which that had DSR > 3.

^b^N, total number of sampled fields.

^c^n, total number of roots evaluated for severity of root rot symptoms.

Among years, means in a row followed by different letters do not significantly differ (Tukey-adjusted LSMeans comparisons for incidence data; Kruskal post hoc test for disease severity data (P < 0.05)).

^d^All roots sampled in organic fields 2019 and conventional fields in 2017 and 2019 appeared healthy (DSR < 3).

Among the years, the highest root rot incidence in both management systems was recorded in 2016 (58% organic and 36% conventional fields), followed by 2018 (50% organic and 29% conventional fields). In 2017, all roots collected from conventional fields showed no visible symptoms of rot (mean DSR<3) whereas roots collected from 21% of the organic fields were symptomatic (mean DSR>3). In 2019, all roots collected from both management systems appeared healthy (mean DSR<3).

### Fungal species associated with root infections

Out of a total of 2175 roots analyzed over the four years, 1939 yielded fungal isolates. A total of 4213 *Fusarium* and 490 *Didymella*- like isolates were obtained from the 110 fields. Of these, 49.7% (n=2093) of the *Fusarium* and 91.8% (n=450) of the *Didymella* isolates originated from organically managed fields (N = 53 fields; n = 946 roots), whereas the remaining 50.3% (n=2093) *Fusarium* and 8.2% (n=40) *Didymella* isolates were obtained from conventional fields (N=57; n=993).

Combined over years and management systems, members of the *Fusarium oxysporum* species complex (FOSC; 36% colonized roots), *F. redolens* (34%), members of the *F. solani* species complex (FSSC; 27%) and *F. avenaceum* (23%) were the most frequent. Together, they constituted 74% of all isolates recovered, and occurred in 82 to 94% of the fields. The next most frequent *Fusarium* species were *F. tricinctum*, *F. culmorum* and *F. equiseti* which were isolated from 6% (*F. equiseti*) to 11% (*F. tricinctum*) of the roots. *Didymella pinodella* was recovered from 17% of all roots, but occurred mainly in organic fields with overall isolation frequency of 31%. The latter four species together represented 23% of all isolates recovered and were found in 44 to 54% of the fields over the years. Species isolated at frequencies ≤ 2% included seven *Fusarium* and one *Didymella* spp.: *F. acuminatum, F. graminearum, F. crookwalance, F. torulosum, F. sporotrichioides, F. sambucinum, F. flocciferum* (Šišić et al., 2020) and *D. eupyrena (*syn*. Juxtiphoma eupyrena).*

**Figure 5:**
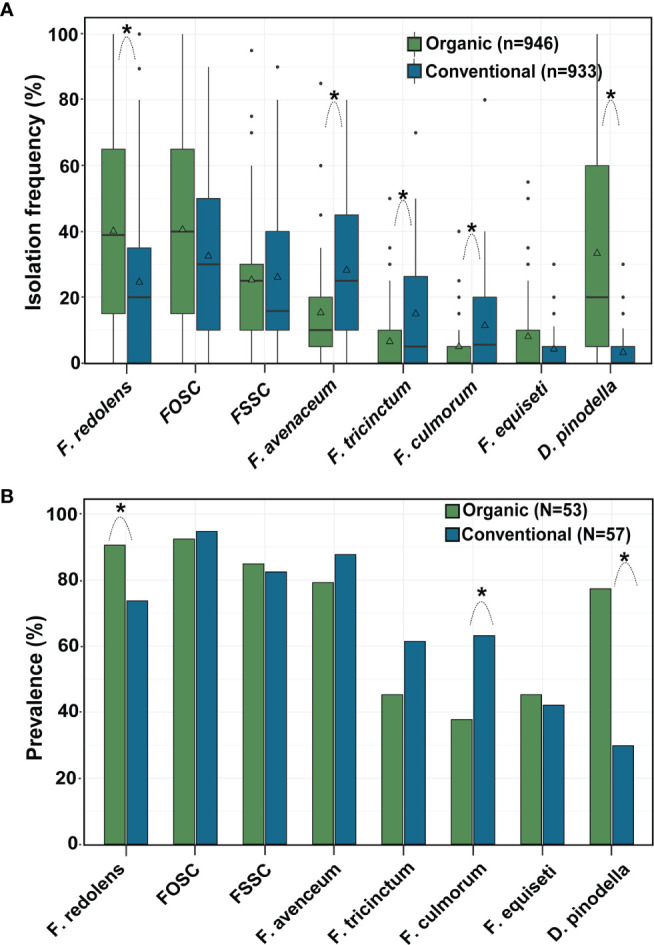
Effect of management system on **(A)** isolation frequency (%) and **(B)** prevalence (% fields yielding a given species) of the eight most common fungal species recovered from faba bean roots. Asterisks indicate significant differences (P < 0.05) between organic and conventional fields for each fungal species separately (Tukey-adjusted pairwise LSMeans comparisons). n = number of roots, N = number of fields evaluated. The horizontal line in the boxplot shows the median value, the bottom and tops of the box the 25th and 75th percentiles and the vertical lines the minimum and maximum values, outliers as single points. Mean values are marked with triangles.

Across years, frequencies of *F. redolens* and *D. pinodella* were significantly higher in roots from organic fields than from conventional fields. In contrast, *F. avenaceum*, *F. tricinctum* and *F. culmorum* more frequently colonized roots in conventionally managed fields (for all comparisons P < 0.01). Overall mean isolation frequencies of the species within the *F. oxysporum* and the *F. solani* species complexes and the mean isolation frequencies of *F. equiseti* did not differ significantly between the management systems (**Figure 5A**).

With few exceptions, the trends observed over the years were similar within years (**Table 4**). For example, during 2016-2018, *F. redolens* was more common in organically grown faba beans roots (31 to 66%) compared with conventionally grown faba beans (17 to 44%) while in 2019, the species occurred only rarely in either management system. *Didymella pinodella* varied considerably in frequency among organic fields depending on the year (15 to 51%) but was consistently low in conventional fields (≤ 6%) (**Table 4**).

**Table 4:** Variation in isolation frequencies (%) of the eight most common fungal species recovered from faba bean roots separated based on sampling year and management system.

System	Year	N^a^	n^b^	*F. redolens*	*F. oxysporum*	*F. solani*	*F. avenaceum*	*F. tricinctum*	*F. culmorum*	*F. equiseti*	*D. pinodella*
Organic	2016	12	240	41.3 ab	40.4 ns	30 ab	20.0 ab	5.0 ab	7.9 ab	16.3 a	15.0 abc
	2017	14	279	30.8 bc	35.1	21.1 ab	17.2 ab	14.0 a	3.6 ab	8.2 ab	40.9 a
	2018	16	317	65.6 a	44.5	32.8 ab	11.4 b	1.9 b	1.9 b	2.2 b	28.7 ab
	2019	11	110	13.6 d	41.8	14.5 ab	13.6 ab	5.5 ab	8.2 ab	7.3 ab	50.9 a
Conventional	2016	14	280	17.1 c	29.6	31.1 ab	37.1 a	21.4 a	9.3 ab	5.0 ab	2.5 cd
	2017	15	298	20.5 bc	28.9	19.1 ab	26.8 ab	11.7 ab	10.4 ab	3.7 ab	2.7 cd
	2018	14	275	44.0 ab	32.0	42.2 a	28.7 ab	7.6 ab	9.5 ab	3.3 ab	1.8 d
	2019	14	140	17.1 bc	40.0	12.1 b	20.7 ab	19.3 a	16.4 a	5.0 ab	5.7 bcd
Overall % colonized roots				34.1	35.8	27.2	22.6	10.6	7.7	6.1	16.8

^a^Number of fields.

^b^Number of roots evaluated.

Means within column (for each species across the years) followed by different letters indicate significant differences (P < 0.05) in single species isolation frequencies across management systems and years (Tukey-adjusted multiple LSMeans comparisons). ns = Non-significant.

*Fusarium avenaceum, F. tricinctum* and *F. culmorum* isolation frequencies varied greatly among years with the latter two species occurring generally less frequently in both management systems (11 to 37% for *F. avenaceum* vs 2 to 21% for *F. tricintum*, and 2 to 16% colonized roots for *F. culmorum*). *Fusarium equiseti* was recovered at low frequencies in both growing systems (2-16%) (**Table 4**) while members of the FOSC and the FSSC were common in isolation frequencies among years and between management systems (**Table 4**).

Pathogen prevalence, *i.e.* the percentage of fields in which the pathogens occurred, followed a similar pattern to the isolation frequencies (**Figure 5B**). Across years*, F. redolens* and *D. pinodella* were more prevalent (P=0.0018) in organic fields (91% for *F. redolens* and 77% for *D. pinodella*) compared with conventional fields (74% for *F. redolens* and 30% for *D. pinodella*) (**Figure 5B** and **Table 5**). Presence of *F. redolens* was considerably lower in conventional fields in 2016 and 2019, while in 2017 and 2018, the prevalence rates of *F. redolens* in organic and conventional fields were more similar (**Table 5**). The prevalence patterns of *D. pinodella* were more variable. Predominantly occurring in organic systems, this species showed moderate to high prevalence rates over the years ranging from 63% (2018) to 93% (2017). In conventional systems, the highest *D. pinodella* prevalence rate was observed in 2017 (47%), the lowest also in 2018 (14%) (**Table 5**).

**Table 5:** Variation in prevalence (% of fields) of the eight most common fungal species recovered from faba bean roots separated based on sampling year and management system.

System	Year	N^a^	n^b^	*F. redolens*	*F. oxysporum*	*F. solani*	*F. avenaceum*	*F. tricinctum*	*F. culmorum*	*F. equiseti*	*D. pinodella*
Organic	2016	12	240	100 a	92 ns	92 ns	100 a	42 ab	58 ab	75 a	75 ab
	2017	14	279	86 ab	93	86	79 ab	79 a	50 abc	57 ab	93 a
	2018	16	317	100 a	88	94	63 b	25 b	19 c	19 c	63 abc
	2019	11	110	73 abc	100	64	82 ab	36 b	27 bc	36 abc	82 ab
Conventional	2016	14	280	57 bc	100	86	93 a	86 a	64 ab	57 ab	29 cd
	2017	15	298	87 ab	80	80	93 ab	53 ab	80 a	40 abc	47 bcd
	2018	14	275	100 a	100	93	93 ab	50 ab	43 bc	43 abc	14 d
	2019	14	140	50 c	100	71	71 ab	57 ab	64 ab	29 bc	29 cd

^a^Number of fields.

^b^Number of roots evaluated.

Means within column (for each species across the years) followed by different letters indicate significant differences (P < 0.05) in single species prevalence rates across management systems and years (Tukey-adjusted multiple LSMeans comparisons). ns = Non-significant.

Although isolated more frequently from roots collected from conventional fields, the overall prevalence rates of *F. avenaceum* and *F. tricinctum* did not differ significantly between the management systems (**Figure 5B**). The prevalence of *F. culmorum* was significantly higher in conventional fields (63%) than in organic (38%) as observed for isolation frequencies.

For the members of the FOSC and FSSC, some variability in the proportion of positive fields was observed similar to the isolation frequencies, but these differences were not significant between the management systems over the years (**Figure 5B**) or during any single year (**Table 5**). Members of the FOSC were consistently found in more than 80% of the fields. The proportion of FSSC positive fields ranged from 64 to 93%. *Fusarium equiseti* prevalence rates varied between 19 and 75% depending on year and growing system (**Table 5**).

### Phylogeny

**Figure 6:**
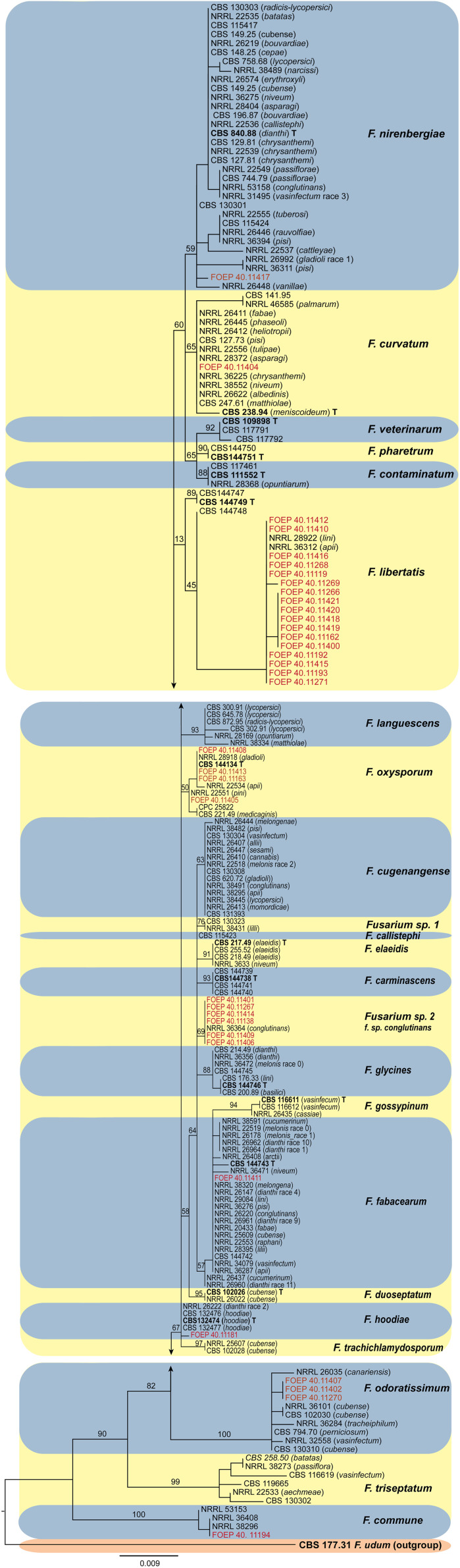
The maximum likelihood (RAxML) tree inferred from the partial *TEF1 alpha* gene sequence alignments of the *Fusarium oxysporum* species complex isolates (FOSC) used in this study. Isolates (i.e. FOEP) are indicated in red. Epi- and ex-type strains are indicated in bold and superscript ‘T’ (Lombard et al., 2018). The scale bar indicates 0.009 expected changes per site. The tree is rooted to *F. udum* (CBS 177.31).

**Figure 7:**
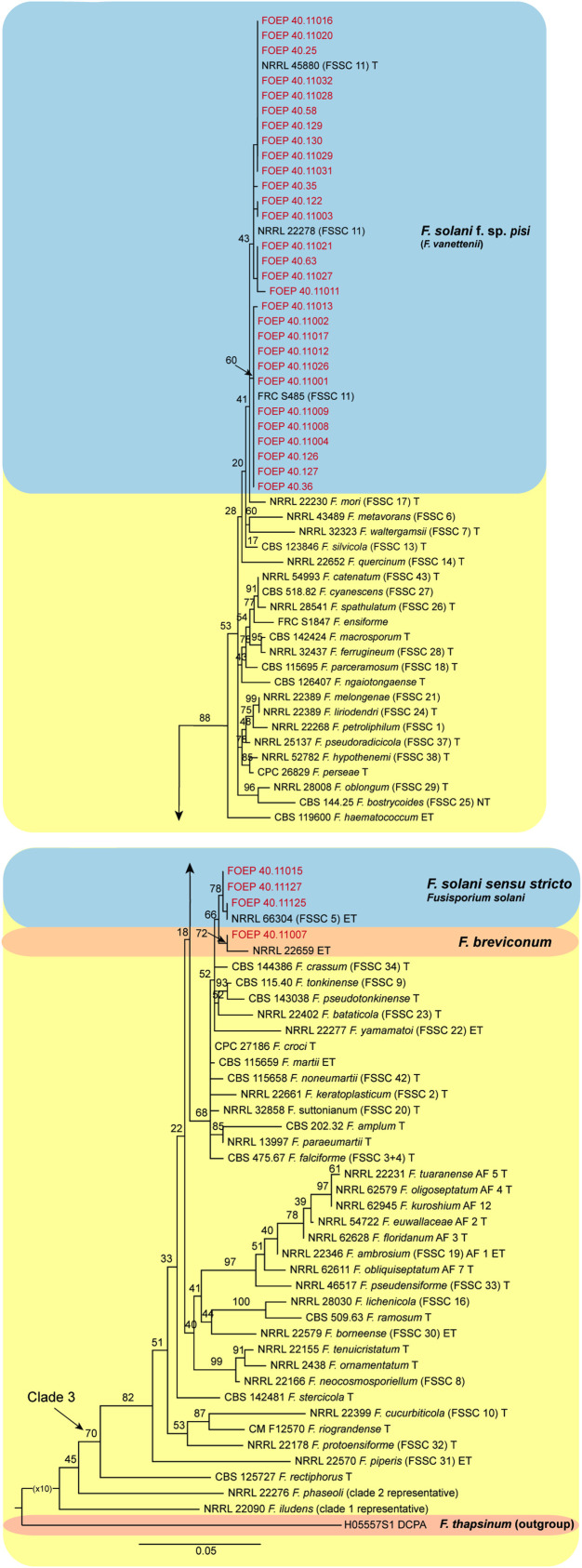
The maximum likelihood (RAxML) tree inferred from the partial *TEF1 alpha* gene sequence alignments of the *Fusarium solani* species complex isolates (FSSC) used in this study. Isolates (i.e. FOEP) are indicated in red. Epi- and ex-type strains are indicated in bold and superscript ‘T’ (O’Donnell et al., 2020; Geiser et al., 2021). The scale bar indicates 0.05 expected changes per site. The tree is rooted to *F. thapsinum* (H05557S1 DCPA).

Phylogenetic analyses inferred from the *TEF1 alpha* gene sequences resolved the phylogenetic positions of the 35 FOSC and 33 FSSC isolates studied in relation to currently recognized species in both species complexes (**Figures 6**, **7**). Both the members of the FOSC and the FSSC varied greatly in morphology, which was reflected in high genetic variability, particularly for the members of the FOSC. Based on the single locus phylogeny, the 35 FOSC isolates were distributed throughout the FOSC clade and belonged to 9 different lineages. The most abundant group comprising 17 isolates was placed in the *F. libertatis* lineage. The second most abundant group, represented by 6 isolates, did not cluster clearly with any of the recently described species within the FOSC. These were most closely related to the previously assigned *F. oxysporum* special form *conglutinans* (NRRL 36364). The results of the *TEF1 alpha* tree topology further revealed 4 isolates matching recently erected epitype specimen (Lombard et al., 2018) and 3 isolates were placed in *F. odoratissimum* linages. In addition, single isolates were placed in the *F. nirenbergie, F. hodiae, F. fabacearum/F. calistephi, F. curvatum* and *F. commune* lineages (**Figure 6**).

The 33 FSSC isolates were placed into three different lineages, all nested within clade 3. Most isolates (n=29) matched *F. pisi* (syn. *F. solani* f. sp *pisi*), the lineage recently renamed to *Fusarium vanettenii* (Geiser et al., 2021). A group of 3 isolates were placed in the *Fusarium solani* sensu stricto lineage, and one isolate matched *F. breviconum* (**Figure 7**).

### Major factors influencing pathogen frequency and faba bean yield

The overall path model from stepwise regression showed a good fit with the data for both organic and conventional fields (**Supplementary Table 8**). In organic fields, yield correlated negatively with *D. pinodella* (*β* = -0.42) and *F. redolens* (*β* = -0.40) frequencies and positively with *F. tricintum* in faba bean roots (*β* = 0.33). In conventional fields, weak but significant negative correlations between yield and FOSC (*β* = -0.20) and *F. culmorum* (*β* = -0.21) were found. Additionally, yield correlated positively with the total precipitation before sowing (*β* = 0.27) and in particular the total precipitation from sowing to root sampling (*β* = 0.48) for conventional faba bean. Overall, these variables accounted for approximately 40% of the yield variation in both management systems (**Supplementary Table 7**).

The frequencies of *F. redolens* in both management systems were positively correlated with the number of days below zero in March (i.e. colder conditions at sowing and plant emergence: path coefficients for organic/conventional system, *β* = 0.44/0.50) and the average temperature measured for the period from sowing to root sampling (*β* = 0.62/0.42). Following this pattern, in organic fields *F. redolens* frequencies was correlated negatively with the average temperature two weeks before sowing (*β* = -0.30). In addition, the frequency of cereals in the preceding five years before sampling (*β* = 0.24) and silty soils (*β* = 0.22) was associated with this pathogen. Cumulatively, these variables explained 55% and 25% of variation in *F. redolens* frequencies in organic and conventional systems, respectively (**Table 6** and **Supplementary Table 7**).

**Table 6:** Summary of the path analysis results showing the main environmental and cropping history factors affecting abundance (isolation frequencies) of major fungal species in roots of organically (Org.) and conventionally (Conv.) grown faba beans.

	Temperature		Precipitation		Crop rotation		Soil		% variance explained	
	Org.	Conv.	Org.	Conv.	Org.	Conv.	Org.	Conv.	Org.	Conv.
*F. redolens*	+ early cold**^1^** + warm season**^2^**				+ cereals		+ silty soils		55	25
FOSC	+ early cold + warm season	- early cold	+ early wet^3^				+ sandy soils		46	26
FSSC	+ early cold			- early wet				+ clay soils	10	34
*F. tricinctum*	- early cold	+ early cold		- early wet					11	20
*F. culmorum*	+ cool season^2^			- early wet + dry season^4^					11	18
*F. avenaceum*	+ cool season								9	
*F. equiseti*	+ cool season		+ early wet		+ cereals				18	
*D. pinodella*	- early cold			+ early wet + dry season	+ legumes	- cereals			33	37

^1^+/- early cold: positive (+) or negative (-) correlation with Average temp. °C (Jan-sowing) and/or, Number of days < 5°C (14 days prior to sowing-sowing) and/or, Number of days <0° in March and/or, Average temp. °C (14 days prior to sowing-sowing) and/or, Number of days < 5°C (sowing-14 days after).

^2^+ warm season: positive correlation with Average temp. °C (sowing-root sampling); +cool season: negative correlation with Average temp. °C (sowing-root sampling).

^3^+/- early wet: positive (+) or negative (-) correlation with Precipitation sum (mm) (28 days prior to sowing-sowing) and/or, Precipitation sum (mm) (14 days prior to sowing-sowing) and/or, Precipitation sum (mm) (sowing-14 days after).

^4^+dry season: negative correlation with Precipitation sum (mm) (sowing-root sampling).

Positive correlations are indicated with ‘+’ and indicate increase in isolation frequencies of a fungal species. Negative correlations are indicated with ‘–’. The original output of the path analysis with standardized path coefficients are given in **Supplementary Table 7**.

The FOSC in both management systems was positively correlated with sandy, slightly acidic soils (*β* = 0.52/0.43). In organic fields, FOSC frequencies correlated positively with the number of days below 5°C two weeks after sowing (*β* = 0.46), the total precipitation from sowing to two weeks after (*β* = 0.21) and the average temperature from sowing to root sampling (*β* = 0.24). Thus, wet and colder conditions up to plant emergence followed by higher temperatures during the main growing season favored this pathogen complex in organic fields similar to what was observed for *F. redolens*. In conventional fields, in addition to the observed correlation with sandy soils, the FOSC frequencies were favored by higher temperatures early in the year (January to sowing*; β* = 0.30). Together, these variables explained 46% and 26% of the FOSC total variance in organic and conventional fields, respectively (**Table 6** and **Supplementary Table 7**).

Similar to *F. redolens* and to the FOSC in organic systems, colder conditions at sowing and/or plant emergence correlated positively with the FSSC frequencies (in conventional system: positive correlation with the number of days below zero in March, *β* = 0.32; in organic system: negative correlation with the average temperatures from January to sowing, *β* = -0.32). The analysis also indicated that low precipitation in the period of plant emergence (from sowing to 2 weeks after; *β* = -0.28) and growing faba bean in clay soils (*β* = 0.31) enhanced FSSC root colonization rates in conventional but not organic fields. Together, these variables explained 10% and 34% of the FSSC total variance in organic and conventional fields, respectively (**Table 6** and **Supplementary Table 7**).

For *F. tricinctum*, 11% of the variance in organic fields was explained by the negative correlation with number of days below 5°C two weeks before sowing (*β* = -0.33), i.e. warmer conditions before sowing favored it. In conventional systems this effect was opposite (*β* = 0.34**)**. In addition, the lower precipitation two weeks before sowing (*β* = 0.23) explained 20% variation in the species root colonization rates data (**Table 6** and **Supplementary Table 7**).

The frequencies of *F. culmorum* in both management systems correlated negatively correlated with the average temperature measured for the period from sowing to root sampling (*β* = -0.28/-0.49). In conventional fields only, there was also a negative correlation of *F. culmorum* frequencies with the total precipitation 4 weeks prior to sowing (*β* = -0.27) and from sowing to root sampling (*β* = -0.38) i.e. colder and drier conditions favored this pathogen. Together, these variables explained 11% and 18% of the *F. culmorum* total variance in organic and conventional fields, respectively (**Table 6** and **Supplementary Table 7**).

The frequencies of *F. avenaceum* and *F. equiseti* could not be related to any of the environmental factors or the cropping history in conventional systems. In organic systems, isolation frequencies of both species correlated negatively with total temperature between sowing and root sampling (*F. avenaceum/F. equiseti*; *β* = -0.31/-0.27). In addition, *F. equiseti* was correlated negatively with the total precipitation two weeks after sowing (*β* = -0.27; as the FSSC) and positively with the frequency of cereals in the preceding five years (*β* = 0.30) similar to *F. redolens*. These variables together explained 9% and 18% of the variation in the *F. avenaceum* and *F. equiseti* data, respectively (**Table 6** and **Supplementary Table 7**).

*Didymella pinodella* in organic fields was positively correlated with frequencies of cool season grain and small seeded legumes in the preceding five years before sampling (*β* = 0.47) and clover and alfalfa for the same period (*β* = 0.31). Conversely, in conventional systems, the frequencies of cereals in the preceding five years before sampling negatively affected *D. pinodella* root colonization rates (*β* = -0.45). Also, *D. pinodella* frequencies were negatively associated with the number of days below zero in March (conventional system: *β* = -0.47) and the number of days below zero 2 weeks prior to sowing (organic system: *β* = -0.27) i.e. opposite to *F. redolens* and FOSC. In conventional systems, *D. pinodella* frequencies were also positively correlated with the total precipitation during the 14 days after sowing (*β* = 0.44) but negatively with the total precipitation from sowing to root sampling (*β* = -0.42) i.e. warmer and wetter conditions at sowing/plant emergence followed by drier growing seasons favored this pathogen in conventional fields. The model explained 33% and 37% of the species variation in organic and conventional system, respectively (**Table 6** and **Supplementary Table 7**).

## Discussion

Root rot incidence was generally low in both management systems especially in 2017 and 2019 when precipitation prior to sowing was low followed by dry growing seasons. Among the 14 *Fusarium* and two *Didymella* species identified, the *F. oxysporum* (FOSC) and *F. solani* (FSSC) species complexes, *F. redolens*, and *F. avenaceum* were present in 82 to 94% of the fields and were most abundant, accounting for 74% of all isolates recovered. Less frequently found *Fusarium* spp. included *F. tricinctum*, *F. culmorum* and *F. equiseti*. The species *D. pinodella* occurred in moderate abundance in organic fields but was much less frequent in conventional fields. *Fusarium redolens* was also more common in roots from organic fields compared with conventional fields. In contrast, *F. avenaceum*, *F. tricinctum* and *F. culmorum* occurred more frequently in conventionally managed fields. Faba bean roots were colonized at similar rates by FOSC, FSSC and *F. equiseti* in both management systems.

There was no difference in the composition nor the frequencies (% colonized roots) of the fungal species in symptomatic and asymptomatic faba bean roots. There were also no effects of the management system (organic vs conventional) on the spectrum of pathogens isolated. Cropping system effects were intricately connected with differences in cropping histories as conventional systems had been devoid of legumes for at least five years in most cases. Cropping history affected the isolation frequencies of a number of species.

The low levels of root rot symptoms observed in some fields may have been due to other factors not assessed in this study. This could have included the presence of other pathogens such as *Pythium* or *Rhizoctonia* spp. (Esmaeili Taheri et al., 2017; Chatterton et al., 2019; Wille et al., 2019) and/or adverse abiotic soil properties such as elevated levels of Fe and Mn contents in the soil which are contributing factors in development of root rot disease (Nayyar et al., 2009). Soil-borne pathogens need to reach a threshold population within the root before causing visible disease symptoms (Gu et al., 2022) and cultural methods fail to reflect pathogen densities in roots. Also, a given root is often colonized by multiple fungal species simultaneously and competitive interactions may have played a role in the generally low symptom expression. For example, we have previously shown that co-occurrence of *F. equiseti* and highly aggressive strains of *F. avenacem* and *D. pinodella* in pea roots can almost completely neutralize detrimental effects of these pathogens (Šišić et al., 2017). Similarly, other beneficial members of root associated microbial communities such as arbuscular mycorrhizal fungi are also known for their ability to reduce biotic stresses (Wille et al., 2019). It is unknown to what extent such interactions played a role in symptom expression. This could be evaluated using, for example, quantitative real-time PCR (qPCR). While qPCR assays targeting all major *Fusarium* species identified in this study including the qPCR for detection and quantification of *D. pinodella* have been developed recently (Zitnick-Anderson et al., 2018; Šišić et al., 2022), these assays were not available when this study was initiated. This study provides solid foundation to further analyze the nature of interactions among the major fungal species isolated as the success of qPCR depends on prior knowledge of the pathogen population targeted. Despite the limitations resulting from the culture based identifications used in our study, taken together, our results indicate a generally high tolerance of faba beans to major root rot pathogens of grain legumes which are a common part of the root microflora of this crop.

### Yield effects

The lack of a clear association between root rot incidence and the major pathogens identified in this study and between root rot incidence and faba bean yield is in line with previous reports pointing to the ability of legume associated *D. pinodella* and *Fusarium* spp. (Rodriguez et al., 2009; Šišić et al., 2018b; Šišić et al., 2022) to infect various hosts without causing visible root rot disease symptoms. Nevertheless, path analysis indicated significant negative correlations of *D. pinodella* and *F. redolens* with yield in organic systems, and significant negative yield effects of FOSC and *F. culmorum* in conventional systems. These results are in line with the hypothesis that there are rarely neutral biological interactions (Schulz and Boyle, 2005). Thus, asymptomatic plant infections likely result from mutually balanced antagonisms between a plants defense system and pathogen virulence factors (Schulz and Boyle, 2005). The positive association of pathogen frequencies with yield reductions in this study could reflect a need for higher investment of the faba bean to maintain a balanced antagonism with the aforementioned pathogens (i.e. absence to low levels of root rot), resulting in lower yields. We have recently demonstrated that *D. pinodella* is highly aggressive on pea causing symptoms and biomass reductions. In contrast, it can colonize wheat roots without causing visible disease symptoms while reducing wheat biomass (Šišić et al., 2022). This is the first instance where predominantly asymptomatic root infections by this pathogen appear to be negatively associated with faba bean yield. The fact that this negative correlation occurred only in organic systems is a result of the differences in rotational history between the management systems as this pathogen was inseparably connected with the frequency of legumes in rotation which constituted only about 5% of the crops in conventional fields compared to 26% of the crops in organic fields (**Figure 4** and **Supplementary Table 5**).

*Fusarium redolens* is commonly isolated from diseased roots of different grain legumes, however its role in the root rot complex is not fully understood. For example, Booth (1971) reported that this pathogen was wide spread in temperate regions causing damping-off, wilts and cortical rots on a variety of non-legume and legume crops including pea and faba bean. A more recent study established *F. redolens* as an important and very aggressive root rot pathogen of pea in northern France causing similar damage as *F. solani* f. sp. *pisi* (Gibert et al., 2022). In contrast, Persson et al. (1997) and more recently Safarieskandari et al. (2020) found *F. redolens* to be a generally weak root rot pathogen based on the *in vitro* pathogenicity assays despite its frequent isolation from symptomatic field pea and lentil roots (Chatterton et al., 2019). In our study, it is possible, however, that asymptomatic infections with *F. redolens* could have affected yields opportunistically when the plants were stressed by cold conditions in early crop growth stages followed by warm and dry conditions. There is a need to more precisely characterize the interactions of this pathogen, faba bean yield and environmental variables including other fungal species. This may be particularly important as *F. redolens* is also pathogenic on cereals (Esmaeili Taheri et al., 2011; Yegin et al., 2017; Gebremariam et al., 2018) which are common part of crop rotations.

In contrast to negative yield effects of *D. pinodella* and *F. redolens*, higher abundance of *F. tricintum* in faba bean roots was positively associated with faba bean yield in organic systems. Although frequently isolated from legume crops (Šišić et al., 2018b; Chatterton et al., 2019), *F. tricinctum* is mainly associated with the Fusarium Head Blight (FHB) complex of small grain cereals in Europe and North America (Uhlig et al., 2007). Our previous research (Šišić et al., 2018b) indicated that this species was only a weak pathogen on pea where root colonization even resulted in increased pea biomass. In contrast, Yan and Nelson (2020) recently reported that *F. tricinctum* is an important soybean root rot pathogen. Due to apparent positive yield effects observed, however, further studies are recommended to better understand the role of this species in the faba bean root rot complex.

In contrast to organic systems, higher abundance of FOSC and *F. culmorum* in faba bean roots led to yield depressions in conventional fields, possibly reflecting cropping system-driven differences in overall soil properties. *Fusarium* root rot of faba bean caused by multiple *Fusarium* species including *F. culmorum*, and *Fusarium* wilt in particular caused by *F. oxysporum*, are among the most destructive diseases of this crop worldwide (Sillero et al., 2010; Lv et al., 2020). However, it is important to note that, in contrast to organic systems, faba bean yields in conventional systems were less affected by the pathogens and depended more on the total precipitation.

### Cropping history effects on main root associated fungal species

Almost no legumes had been grown in the 5-11 years preceding the conventional faba beans surveyed in this study while grain legumes (mostly pea and faba bean) and also clover and alfalfa had almost always been present during this period in the organic fields sampled. This difference in rotational histories was strongly correlated with the occurrence of *D. pinodella*, which was ubiquitous in organic fields but much rarer or even absent in conventional fields. (Bainard et al., 2017) reported similar results with pea intensified rotations leading to substantial increase in abundance of this pathogen in soil and pea roots.

It is likely that the higher frequencies of legumes in organic rotations also contributed to higher abundance of *F. redolens* in organic systems compared to conventional as abundance of this pathogen in soil has been shown previously to increase substantially following intensified grain legume rotations (Bainard et al., 2017). The positive correlation of this pathogen with the frequency of cereals in organic systems is not surprising, however, as *F. redolens* is also a wheat (Esmaeili Taheri et al., 2011; Gebremariam et al., 2018) and barley (Yegin et al., 2017) pathogen.

The higher relative abundance of *F. avenaceum*, *F. tricinctum* and *F. culmorum* in conventionally grown faba beans is likely related to the generally higher ratio of cereals in conventional rotations despite the absence of clear correlations. In most conventional fields, cereals were grown three to five times in the five years preceding faba beans. The three species are major small grain cereal and maize pathogens frequently associated with ear, stem and root rots and responsible for pre-harvest mycotoxin contaminations (Bottalico and Perrone, 2002; Pfordt et al., 2020). Among the three species, *F. avenaceum* is the most important and wide-spread. It is an opportunistic pathogen especially in the absence of organic matter (Baćanović-Šišić et al., 2018) and the major causal agents of pea and lentil root rot in Canada (Esmaeili Taheri et al., 2016; Chatterton et al., 2019) and pea root rot in the USA (Chittem et al., 2015) in the past 20 years. In Europe, *F. avenacem* is frequently isolated from diseased pea roots but usually at moderate frequencies (Persson et al., 1997; Pflughöft et al., 2012; Baćanović-Šišić et al., 2018; Šišić et al., 2019).

### Effects of environmental conditions on major fungal species identified

Pedo-climatic conditions appeared to be the main drivers for the occurrence of most of the *Fusarium* species identified in this study. Cold conditions at sowing and plant emergence and/or during the vegetative season in particular were found to favor most of the dominant *Fusarium* species identified in this study. These conditions were associated with increased root colonization rates by *F. redolens, F. solani* and *F. culmorum* in both management systems, and the FOSC and *F. equiseti* in organic systems. Sowing into cold soils prolongs seedling emergence favoring early plant infections by these pathogens (Naseri and Marefat, 2011). Although *Fusarium* spp. can infect their hosts at all growth stages, previous research has shown that the timing of infection plays a crucial role in the extent of root rot severity and yield reduction (Papavizas, 1974; Šišić et al., 2017; Navas-Cortés et al., 2020). For example, Šišić et al. (2017) showed that the detrimental effect of *F. avenaceum* on pea root health and biomass depended strongly on the timing of pathogen inoculation. When inoculated at sowing, *F. avenaceum* caused severe wilting resulting in 83% pea biomass loss compared to a non-inoculated control. In contrast, inoculation five days after pea sowing resulted in moderate root rot disease severity with greatly reduced negative effects on pea biomass (-14%). Therefore, vigorous seeds are likely to rapidly outgrow the highly susceptible seedling growth stage, reducing the overall risks of *Fusarium* damage. But also higher temperatures e.g. hot weather following sowing and/or in the period from sowing to root sampling can favor root infections e.g. by *F. redolens* which was more severe in both management systems in hot and dry seasons. The opposite was observed for *F. culmorum* in both management systems and *F. avenaceum* and *F. equiseti* in organic systems. Their root colonization rates were favored by cooler growing season. The precipitation effects were highly variable and mostly management system and *Fusarium* species specific. While *Fusarium* spp. are able to adapt to various ranges of environmental conditions, the competitive advantage of each species in the faba bean root rot complex will vary depending on the site specific pedo-climatic conditions (Yergeau et al., 2009). Furthermore, abiotic plant stress (i.e. the crop defense response) seems to have an important impact on the susceptibility of faba beans to *Fusarium* infections.

In contrast to *Fusarium* spp., warmer conditions at sowing/plant emergence favored *D. pinodella* in both management systems. In addition, although this species occurred rarely in conventional systems, *D. pinodella* frequencies were positively correlated with warmer and wetter conditions at sowing/plant emergence followed by drier growing seasons. The positive effect of drier conditions on *D. pinodella* root colonization rates and especially the high incidence of this pathogen in 2019, the driest sampling year in this study, are in contrast with what has been reported in the *D. pinodella-*pea system where infections are primarily favored by wet and humid conditions during the main growing season (Bretag et al., 2006; Esmaeili Taheri et al., 2016). These results suggest that short periods of wet conditions are sufficient for infections by this pathogen and also point to its opportunistic nature where abiotic plant stress (e.g. lack of precipitations) can enhance colonization process once the primary infections occur.

Our observations that the frequency of the FOSC was correlated positively with sandy soils characterized by lower pH supports previous reports that sandy soils and lower pH are more conducive for this pathogen and contribute to increased root rot and wilt incidence in a range of different crops, including chickpea, banana, flax, carnations, watermelon, tomato and marigold (Scher and Baker, 1980; Amir and Alabouvette, 1993; Singh et al., 2017; Orr and Nelson, 2018; Saeedi and Jamali, 2021). Some of these studies also showed that silty and clay soils and/or increasing soil pH were often suppressive to *Fusarium* wilt development. We also found a weak but statistically significant association of *F. redolens* with organic silty soils in this study. (Saeedi and Jamali, 2021) recently reported that *F. redolens* is a very aggressive pathogen of chickpea in Iran where *F. oxysporum* dominated on sandy soils while *F. redolens* was highly correlated with low sand and organic matter among others. In addition, the positive correlation between the FSSC frequencies in conventional system and silty-clay soils with generally high SOM content (*i.e.* fields in soil cluster 3) suggest that the FSSC members are highly competitive saprophytes.

### Genetic diversity among *F. oxysporum* and *F*. *solani* isolates

The observation that the members of the FOSC and the FSSC are important components of the faba bean root rot complex both in organic and conventional fields is consistent with many previous findings (Chittem et al., 2015; Esmaeili Taheri et al., 2016; Šišić et al., 2018a; Šišić et al., 2018b; Chatterton et al., 2019). The wide-spread occurrence of the FOSC and FSSC over a range of soil and environmental conditions observed in this study indicates a high adaptability of both species complexes to a range of pedo-climatic and environmental conditions, which may in turn indicate high genetic diversity. The high genetic variability among the 35 F*. oxysporum* isolates from a single host observed in this study came as a surprise, however. With the exception of *F. curvatum, F. nirenbergie, F. oxysporum* and *F. fabacearum* lineages which have been previously associated either with faba bean or other legumes, the remaining five lineages including the most abundant *F. libertatis* (17/35 isolates) have not been associated with any legume host previously. Further analysis is required to determine their role in the faba bean root rot complex.

In contrast to FOSC, 29 of the 33 FSSC isolates analyzed matched *F. pisi* (syn. *F. solani* f. sp. *pisi*; *Fusarium vanettenii*), and a group of 3 isolates were placed in *Fusarium solani sensu stricto* lineage, and one isolate matched *F. breviconum*. These results confirm the common association of *F. pisi* with various legumes and its ability to occupy diverse ecological niches (Šišić et al., 2018a). More recently, Safarieskandari et al. (2020) demonstrated a high level of aggressiveness of *F. pisi* to faba beans. It is also important to note that, while the phylogenetic analysis generally confirmed a good resolution power of the *TEF1 alpha* locus in *Fusarium*, poor bootstrap support for some lineages within the FOSC was observed. These results were expected however, due to the single locus analysis and were consistent with the results of the *TEF1 alpha* tree topology reported for the FOSC (Lombard et al., 2018) and for the FSSC (Šišić et al., 2018a; Geiser et al., 2021). Work is on-going to obtain additional sequence data from more loci and also aggressiveness tests to better understand genetic variation and the role of the collected isolates in the faba bean root rot complex.

## Conclusions

This four-year survey provides the first comparative documentation on the prevalence and frequency of *Fusarium* and *Didymella* species associated with faba bean roots in organic and conventional fields in Germany. By covering a wide range of pedoclimatic and field history conditions under conventional and organic production systems, it was possible to develop inferences about the main drivers currently influencing the pathobiome community on faba beans. Pedoclimatic conditions were the main drivers of the *Fusarium* communities, whereas *D. pinodella* was primarily influenced by the presence of grain legumes in the recent cropping history. This led to the dominance of this pathogen in organic systems, almost certainly because of the higher frequency of legumes in organic rotations. The role of *F. libertatis* and the other FOSC members identified that had not previously been associated with root rot of grain legumes needs to be examined. This study indicated that several major root rot pathogens of grain legumes may asymptomatically colonize faba bean roots as had been reported previously for other crops (Šišić et al., 2018b; Šišić et al., 2022). The study indicated that faba bean yields in organic systems apparently were affected by asymptomatic root infections by *D. pinodella* and *F. redolens* whereas yields in conventional systems depended more on the total precipitation during the main growing season. As described in the introduction, the new protein strategy of the EU has encouraged many conventional farmers to adopt grain legumes since 2012 and our survey shows that most conventionally grown faba beans were grown for the first time in many years. An overall increase of grain legume production under conventional conditions will likely change their health status, however, as has already been observed in Canada (Bainard et al., 2017; Niu et al., 2018).

## Data availability statement

The original contributions presented in the study are included in the article/**Supplementary Files**. Further inquiries can be directed to the corresponding author.

## Author contributions

AŠ, HS, JŠ, MF study design/methodology/investigation. AŠ data analysis/drafted the manuscript. AŠ, MF resources/funding acquisition. All authors contributed to the article and approved the submitted version.
